# Suicide Methods in Asia: Implications in Suicide Prevention

**DOI:** 10.3390/ijerph9041135

**Published:** 2012-03-28

**Authors:** Kevin Chien-Chang Wu, Ying-Yeh Chen, Paul S. F. Yip

**Affiliations:** 1 Department of Social Medicine, School of Medicine, College of Medicine, National Taiwan University, 2F Medical Humanity Building, No. 1, Section 1, Ren-Ai Road, Zhong Zheng District, Taipei 10051, Taiwan; Email: ccwu88@ntu.edu.tw; 2 Department of Psychiatry, National Taiwan University Hospital, No. 1, Changde Street, Zhong Zheng District, Taipei 10048, Taiwan; 3 Taipei City Psychiatric Center, Taipei City Hospital, 309 Songde Road, XinYi District, Taipei 11080, Taiwan; 4 Institute of Public Health and Department of Public Health, National Yang-Ming University, No. 155, Section 2, Linong Street, Bei Tou District, Taipei 11221, Taiwan; 5 Department of Social Work and Social Administration, University of Hong Kong, Pokfulam, Hong Kong; Email: sfpyip@hku.hk; 6 Hong Kong Jockey Club Center for Suicide Research and Prevention, University of Hong Kong, Pokfulam, Hong Kong

**Keywords:** suicide, suicide method, Asia, trend, age, sex, technology, information, media, culture, religion, economic, public health

## Abstract

As the largest continent in the World, Asia accounts for about 60% of World suicides. Preventing suicide by restricting access to suicide methods is one of the few evidence-based suicide prevention strategies. However, there has been a lack of systematic exploration of suicide methods in Asian countries. To amend this shortage, the current review examines the leading suicide methods in different Asian countries, their trend, their age- and sex- specific characteristics, and their implications for suicide prevention. In total, 42 articles with leading suicide methods data in 17 Asian countries/regions were retrieved. The epidemiologic characteristics and recent trends of common suicide methods reflect specific socio-cultural, economic, and religious situations in the region. Common suicide methods shift with the introduction of technologies and constructions, and have specific age- or sex-characteristics that may render the restriction of suicide methods not equally effective for all sex and age sub-groups. Charcoal burning, pesticide poisoning, native plant poisoning, self-immolation, and jumping are all prominent examples. In the information society, suicide prevention that focuses on suicide methods must monitor and control the innovation and spread of knowledge and practices of suicide “technologies”. It may be more cost-effective to design safety into technologies as a way of suicide prevention while there is no rash of suicides yet by the new technologies. Further research on suicide methods is important for public health approaches to suicide prevention with sensitivity to socio-cultural, economic, and religious factors in different countries.

## 1. Introduction

Suicide is one of the leading causes of death in the World. Approximately one million people commit suicide each year, or about one life lost every 40 seconds [1]. As the largest continent in the World, Asia accounts for about 60% of World suicides, with China, India, and Japan accounting for about 40% of the World’s suicides [2]. However, probably due to religion, socio-cultural factors, or lack of reliable death certification procedure, the extent of underestimation of suicide rate in this region has been a problem, especially in China and India [3]. In fact, there are countries in Asia that do not systematically report suicide statistics at all and as such, the problem of suicide may be more severe than what the numbers show.

Preventing suicide by restricting access to suicide methods is one of the few evidence-based suicide prevention strategies [4]. Effective suicide prevention requires good studies on the use of suicide methods in different countries. Although research into suicide methods in Western countries (Europe and the U.S.) has been abundant, a more thorough understanding of the use and impact of suicide methods is still lacking, especially in Asia. As shown above, suicides in Asia comprise of the majority of suicides in the World. Thus it is necessary to inquire into the suicide methods patterns in Asia as a preliminary engagement with the tasks of suicide prevention. Also, the focus on Asia is a good beginning to explore further the importance of sociocultural differences in suicide prevention. 

Suicide methods differ prominently in Western and Asian countries. For example, the use of firearms is the favored suicide method in many Western countries, but not in Asia [5]. In contrast, pesticide ingestion, charcoal burning, and self-immolation are all unique leading suicide methods in Asia. Hanging can be found to be a leading suicide method both in the East and the West. Intensive research for more “customized” suicide prevention policies in the region is warranted. 

However, Asian countries are not homogenous under the “Asia umbrella”. They vary greatly in terms of religious, socio-cultural, economic, and legal backgrounds [3,6,7]. Within Asia, suicide methods vary across countries. The current review delineates the leading suicide methods in different Asian countries and the trend of their changes. The gender and age characteristics of different suicide methods were also examined if relevant information is available. Based on the above findings, a conceptual scheme that goes beyond the traditional restriction of physical access to suicide methods is being proposed. Specifically, it is posited that to effectively prevent suicide, the mechanisms of how knowledge on suicide method spread and settle in sociocultural structures should also be explored [8,9]. 

## 2. Methods

Using “suicide”, “method”, “means”, “Asia”, and the names of Asian countries as the key words, literature related to suicide methods in different Asian countries were searched on PubMed, PsycINFO, and Google Scholar. Only literature from 1990 to September 2011 was included. Papers not presenting a ranking of suicide methods were excluded. Books and book chapters obtained through independent search or mentioned in the reference list of the reviewed papers were included if they matched the selection criteria. The search yielded 42 papers.

Because many Asian countries (e.g., China and India) did not have a suicide surveillance system for the entire population, suicide data are only available in some localities. Hence, whole population data or data based on local samples were both presented. The most updated suicide rate figures, common methods of suicide, and age- and sex-specific patterns of suicide were presented. Whenever available, the changing patterns of suicide trends in relation to method used were also presented.

## 3. Results

In total, 17 Asian countries/regions were included in the review. These were Bahrain, Bangladesh, China, Hong Kong, India, Iran, Japan, South Korea, Malaysia, Pakistan, Philippines, Saudi Arabia, Singapore, Sri Lanka, Taiwan, Thailand, and Turkey (Table 1). According to available information, Bangladesh, China, Hong Kong, Japan, South Korea, Sri Lanka, and Taiwan had relatively higher suicide rates (>13.0/100,000), with Bangladesh having the highest (39.6/100,000). However, it should be noted that the data on Bangladesh was derived from one local area involving women aged 10–50 years only. 

In countries where whole population suicide rates were available, South Korea had the highest rate (31.0/100,000). In addition to the Philippines, Muslim countries such as Iran, Pakistan, Saudi Arabia, and Turkey had relatively lower suicide rates (<6.5/100,000), with Iran having the lowest (male: 0.3/100,000; female: 0.1/100,000). Due to stigmatization and legal sanctions against suicide, under-reporting of suicide rates in these Muslim countries may partly contribute to the extremely low rate [6,10]. Overall, majority of the Asian countries had lower male-to-female suicide gender ratios (<2.0) compared to their western counterparts [7]. These countries included Bahrain, China, Hong Kong, India, South Korea, Philippines, Singapore, and Turkey. China has the lowest suicide gender ratio (0.88 in 1999), whereas Iran (3.0 in 1991), Pakistan (2.9–4.0 in the period 1991–2006), and Thailand (3.2 in 2002) had higher gender ratios compatible to Western figures. Japan (2.7 in 2009) and Taiwan (2.1 in 2010) were in the middle.

**Table 1:** Suicide methods in Asia.

Countries	Suicide rate (per 100,000 population) Total, Male, Female (Year(s))	Year(s) of observation	Population observed	First common suicide method	Second common suicide method	Third common suicide method	Trend in suicide methods
Bahrain	Total: N/A Male: 4.0 Female: 3.5 (2006) Whole country (WHO)	1995–2004 [11]	General population: registered suicide cases (N = 304) at the Ministry of Interior	Hanging (92.8%)	N/A	N/A	N/A
Bangladesh	Total: 39.6 Male: N/A Female: N/A (1983–2002) Jessore area [6]	1996–1997 [12]	Women of 10–50 years served by health and public facilities	Poisoning (75%)	Hanging or suffocation (22%)	N/A	N/A
China	Total: 13.9 Male: 13.0 Female: 14.8 (1999) Whole country (WHO)	1998–2000 [13]	National sample of suicides (N = 514)	Poisoning with agricultural chemicals or rat poisons (62%)	Hanging (20%)	Poisoning with other substances (7%) Drowning (5%)	N/A
Hong Kong	Total: 14.6 Male: 19.0 Female: 10.7 (2009) Whole country (WHO)	1990–2007 [14]	Whole population in Hong Kong	Jumping (46%) in 2003	Charcoal burning (25%) in 2003	Hanging (19%) in 2003	Suicide with charcoal burning was first described in 1998, and rapidly became the second leading suicide method [15]
India	Total: 10.5 Male: 13.0 Female:7.8 (2009) Whole country (WHO)	a. 1994–1999 [16]	a. Local cohort, Kaniyambadi (N = 609)	a. Poisoning (45%)	a. Hanging (41%)	a. N/A	N/A
		b. 1996–2005 [17]	b. Hospital-based autopsy reports, Northern area (N = 1,421)	b. Poisoning (47.1%), half of which was aluminium phosphide	b. Self-immolation (39.5%)	b. Hanging (8.2%)	
		c. 1996–2005 [18]	c. Local cohort, Kerala (N = 385)	c. Hanging (50%)	c. Poisoning (30%)	c. Drowning (9%)	
		d. 1997–1998 [19]	d. Population-based verbal autopsy, rural Tamil Nadu (N = 3,429)	d. Poisoning (46.6%)	d. Hanging (35.8%)	d. Self-immolation (15.6%)	
		e. 2000–2002 [20]	e. Local cohort, Tamil Nadu	e. Hanging (49%)	e. Organo-phosphates (40%)	e. N/A	
		f. 2000–2003 [21]	f. Hospital-based autopsy reports, West coastal region (N = 539)	f. Hanging (36.9%)	f. Poisoning (34.7%)	f. Drowning (16.0%) Self-immolation (10.0%)	
		g. 2000–2003 [22]	g. Hospital-based autopsy reports, Eastern area (N = 588)	g. Hanging (32.6%)	g. Poisoning (30.6%), one third of which was native plant poisoning	g. Self-immolation (18.7%) Railway run over (17%)	
		h. 2003 [23]	h. Government of India Suicide data	h. Poisoning (37.1%)	h. Hanging (28.4%)	h. Self-immolation (9.7%)	
Iran	Total: N/A Male: 0.3 Female:0.1 (1991) Whole country (WHO) Total: 9.4 Male: N/A Female: N/A (2003) Whole population [24]	a. 1992–2005 [25]	a. University-based statistics in Ilam Province (N = 652)	a. Drugs (44.7%)	a. Burning (25.2%)	a. Toxin (21.5%)	N/A
		b. 2003–2004 [26]	b. Population-based surveys in 23 provinces and 13 studies from “the mien of health in Iran” (N = 4,267)	b. Hanging (34.2%)	b. Self-immolation (27%)	b. Poisoning (18.8%)	
Japan	Total: 24.4 Male: 36.2 Female: 13.2 (2009) Whole country (WHO)	a. 1994 [27]	a. N/A	a. Hanging (55.6%)	a. Jumping (10.4%)	a. Drowning (8.5%) Poison or drug overdose (5.6%)	Increased proportions of hanging and gas poisoning in all suicides Poisoning has replaced jumping as the second leading suicide method.
		b. 1995–2006 [28]	b. Whole population, based on OECD Health Data 2009	b. Hanging (N/A)	b. Other poisoning (N/A) which includes gas poisoning (11.7%)	b. Jumping (N/A)	
		c. 1999 [29]	c. Whole population (N = 31,413)	c. Hanging (70.4%)	c. Jumping from a high place (8.3%)	c. Gases (6.6%) Pesticide (2.4%) Drugs (1.2%)	
		d. 2002–2003 [30]	d. Population-based data in Okayama (N = 824)	d. Hanging (63%)	d. Poisoning by other substances including gas (14%)	d. Drowning (6.3%) Jumping (4.5%)	
South Korea	Total: 31.0 Male: 39.9 Female: 22.1 (2009) Whole country (WHO)	a. 1995, 2000 [31]	a. Whole population	a. Poisoning (43%)	a. Hanging (33%)	a. N/A	The pattern of suicide method did not change much Increased proportions of Hanging and Jumping in all suicides Before 2003, hanging was the leading suicide method [32]
		b. 2003 [32]	b. Whole population	b. Pesticide/ chemicals (40.4%)	b. Hanging (33.9%)	b. Jumping (15%)	
		c. 2004–2006 [33]	c. Whole population, data offered by national agencies	c. Hanging (44.9%)	c. Drug/pesticide intoxication (35.3%)	c. Falling from height (14.4%)	
		d. 2006 [28]	d. Whole population, based on OECD Health Data 2009	d. Hanging (N/A)	d. Poisoning with a majority of pesticide poisoning (N/A)	d. Jumping (N/A)	
Malaysia	N/A	a. 1995–1998 [34]	a. Kuala Lumpur, autopsy reports (N = 84)	a. Hanging (36.9%)	a. Poisoning (35.7%)	a. Falling from height (15.5%)	In Kuala Lumpur, the proportion of jumping suicide increased
		b. 1999 [35]	b. Kuala Lumpur, autopsy reports (N = 76)	b. Poisoning (39%)	b. Hanging (34%)	b. Jumping (22%)	
		c. 2000–2004 [36]	c. Kuala Lumpur, autopsy reports (N = 251)	c. Hanging (43%)	c. Fall (34%)	c. Poisoning (15%)	
		d. 2007–2009 [37]	d. Penang Island, autopsy reports (N = 138)	d. Jumping from height (47.1%)	d. Hanging (34.1%)	d. Drowning (10.9%)	
Pakistan	Total: 0.43–2.86 Male: 0.6–5.2 Female: 0.2–1.8 (1991–2006) (Khan and Haider, 2008)	a. 1985–1999 [38]	a. Police data from Sindh Province (N = 2,568)	a. Poisoning by organophosphate (N/A)	a. Hanging (N/A)	N/A	N/A
		b. 1985–2006 [39]	b. Summary from 7 studies (N = 5,394)	b. Poisoning (34%)	b. Hanging (26%)	b. Firearms (16%) Drowning (11%) Self-immolation (5%)	
		c. 2003 [10]	c. First 100 suicides in Karachi police reports that year	c. Hanging (40%)	c. Poisoning (26%)	c. Firearms (15%) Self-immolation (10%)	
Philippines	Total: 2.1 Male: 2.7 Female: 1.7 (1993) Whole country (WHO)	Years N/A [40]	Summary from available studies	Hanging (N/A)	Shooting (N/A)	Ingestion of chemicals including organophosphate (N/A)	N/A
Saudi Arabia	Total: 1.1 Male: N/A Female: N/A (1986–1995) [41]	1986–1995 [41]	Cases from Medical-Legal Center, Dammam (N = 221)	Hanging (63%)	Jumping (12%)	Gunshot (9%)	N/A
Singapore	Total: 10.7 Male: 12.9 Female: 7.3 (2006) Whole country (WHO)	2000–2004 [42]	Whole population	Jumping (72.4%)s	Hanging (16.6%)	Poisoning (5.9%)	Before 1960–1964, hanging still was the leading suicide method; since 1980–1984 jumping has been the leading suicide method.
Sri Lanka	Total: 21.6 Male: N/A Female: N/A (1996) Whole country (WHO) Total: N/A Male: 37.3 Female: 9.7 (2005) Whole country [43]	a. 1975–2005 [43]	Whole population	Pesticide poisoning (N/A)	Hanging (N/A)	Drowning (N/A) Burning (N/A)	The suicide rate of pesticide poisoning decreased prominently after the 1995 and 1998 pesticide regulations [43] The suicide rate of hanging gradually increased after the above pesticide regulation [43]
		b. 2006 [44]	b. Coroner’s court report in Colombo(N = 151)	b. Poisoning (44%)	b. Burning (34%)	b. Hanging (11%)	
Taiwan	Total: 16.8 Male: 22.7 Female: 10.9 (2010) Whole country (Taiwan Suicide Prevention Center)	a. 1970–1980s [45]	a. Whole population	a. Solids/liquids poisoning (N/A)	a. Hanging (N/A)	a. Others (N/A)	Rapid escalation of charcoal burning as the leading suicide method, especially in the urban areas Suicide rate by jumping also escalated rapidly in 1990–2000s
		b. 1990s [45]	b. Whole population	b. Hanging (N/A)	b. Solids/liquids poisoning (N/A)	b. Others (N/A)	
		c. 1995–2004 [46]	c. Whole population	c. Hanging (43.5%)	c. Solids/liquids poisoning (26.4%)	c. Poisoning by other gases (9.4%) Jumping from heights (9.4%)	
		d. 2005 [45]	d. Whole population	d. Hanging (N/A)	d. Other gases (mainly charcoal burning) (N/A)	d. Solids/liquids poisoning (N/A)	
		e. 2002–2008 [47]	e. Whole population	e. Hanging (30–31%)	e. Charcoal burning (19–29%)	e. Pesticide poisoning (12–15%)	
Thailand	Total: 7.8 Male: 12.0 Female: 3.8 (2002) Whole country (WHO)	a. 1998–2003 [48]	a. Whole population	a. Hanging (N/A)	a. Poisoning with agricultural chemicals (N/A)	a. Other substance poisoning (N/A) Drugs poisoning (N/A) Firearm (N/A)	Poisoning with agricultural chemicals increased in absolute terms and proportion from 1998 to 2003 [48] Since 1997, the rate of female suicide by hanging began to surpass that by self-poisoning [49] In 2001–2005, ingesting agricultural chemicals took the place of other substance intoxication as the second leading suicide method [49]
		b. 2001–2005 [49]	b. Whole population	b. Hanging (58.3%; Male: 60.9%, female: 50.0%)	b. Poisoning with agricultural chemicals (Male: 14.8%; female: 24.5%)	b. Drugs poisoning (Male: 4.4%; female: 7.9%)	
Turkey	Total: 3.7 Male: 4.8 Female: 2.7 (2005) Whole country (Turkish Statistic Institute) [50]	a. 1984–2004 [51]	a. Autopsy cases in Trakya, Turkey (N = 137)	a. Hanging (40.1%)	a. Firearm (21.1%)	a. Poisoning (19.7%)	The pattern of leading suicide methods did not change in decades
		b. 1990–2000 [52]	b. Whole population	b. Hanging (48.2%)	b. Firearm (19.2%)	b. Chemicals (14.8%) Jumping from height (11.0%)	
		c. 1996–2005 [53]	c. Autopsy cases in Bursa, Turkey (N = 955)	c. Hanging (51.6%)	c. Firearm (26.3%)	c. Insecticide poisoning (10.1%)	
		d. 1996–2005 [50]	d. Whole population	d. Hanging (44.8%)	d. Firearm (22.2%)	d. Chemicals (16.3%) Jumping from height (10.3%)	

### 3.1. Leading Suicide Methods in Different Asian Countries

Hanging was the most common suicide method in nine out of the seventeen countries/regions reviewed (*i.e.*, Bahrain, Iran, Japan, South Korea, Philippines, Saudi Arabia, Taiwan, Thailand, and Turkey) (Table 1) [11,26,27,28,29,30,40,41,45,46,47,48,49,50,51,52,53]. Solid/liquid poisoning (mostly using pesticides) was the leading suicide method in three countries: China, Pakistan, and Sri Lanka, which may be related to their agricultural activities and thus, the easy access [54]. Jumping was the leading suicide method in Hong Kong and Singapore [14,42], where around 80% of the residents live in high rise buildings that provide access to this very fatal suicide method [55]. Three countries had uncertain or mixed results: Bangladesh (solid/liquid poisoning, women aged 10–50), India (hanging and solid/liquid poisoning in local results), and Malaysia (hanging and jumping in local results) (Table 1). 

Overall, hanging and solid/liquid poisoning (pesticide) were dominant methods of suicide in Asia. Certain methods rarely reported in the West were, however, very common in some Asian countries. For example, self-immolation was the second most common suicide method in Iran and India (in some reports) and charcoal burning suicide was the second most common method in Taiwan and Hong Kong (Table 1). Drowning was found to be the third leading suicide method in localities of India (9–16%) [18,23], Japan (6–8%) [27,28], Malayasia (10.9%) [35], and Pakistan (11%) [39]. Railway runover was the fourth leading suicide method in an eastern area of India [22]. It is worthy of follow-up whether drug poisoning, railway runover, and gassing in motor vehicles will emerge to become the overall leading suicide methods in the future.

In 16 European countries investigated by Värnik *et al.*, hanging, drugs poisoning, jumping, firearms, other poisoning, jumping or lying before moving objects and drowning are common methods of suicide [56], However, firearms and jumping before a moving objects usually are not leading suicide methods in most studied Asian countries. Furthermore, leading suicide methods in Asia, such as pesticide poisoning, charcoal burning and self-immolation, are so rare that they are not even listed separately in the European study. 

### 3.2. Trend of Changes in Leading Suicide Methods

Trend data on methods of suicide were available only in nine countries/regions. In Turkey and in Kuala Lumpur, Malaysia, the ranking of common suicide methods had been stable over their observed periods. In South Korea, the ranking of the leading suicide methods had been consistent, though the proportions of hanging and poisoning increased over the years. 

The ranking of common suicide methods did change in the other six countries/regions. In Hong Kong, the first case of charcoal burning suicide by inhaling lethal carbon monoxide was in 1998 [57], but in 2003, charcoal burning had become the second leading suicide method there [14]. A similar situation happened in Taiwan. In the 1970s and 80s, solid/liquid poisoning was the leading suicide method. In the 21st century, charcoal burning had overtaken solid/liquid poisoning as the second most common method [45]. Suicide rates in both Hong Kong and Taiwan increased in the 1990s, and then started to diminish around 2005 for Hong Kong [58] and in 2006 for Taiwan [59]. Literature has attributed the increases to the wider adoption of charcoal burning as the suicide method in both regions [15,47,60,61]. Hong Kong Government has been promoting the replacement of charcoal barbecue with the electric grills [62]. Some universities and non-governmental organizations in Hong Kong have succeeded in convincing supermarkets to display warning labels on the charcoal packs [63]. An exploratory controlled trial has shown that putting away the charcoal packs behind the counters of retail outlets did reduce the charcoal burning suicide rate in the locality that adopted the measure [64]. On the other hand, in Taiwan initiatives of printing warning signs on the charcoal packs and gatekeeper education of charcoal retail clerks have been ongoing [59]. Whether these measures will reduce the overall charcoal burning suicide rate is worthy of further analysis. 

In Japan, hanging has been the dominant method of suicide accounting for more than 60% of the suicide deaths [27,28]. An increasing trend of poisoning suicide (mainly gas inhalation) has been observed in the past decade and has replaced jumping as the second common suicide method, accounting for approximately 10% of suicides nowadays. Suicide rate in Japan started to escalate in 1990s and the trend continued as in 2009 especially in males [65]. The time period overlapped with the increased incidence of charcoal burning suicide (much more frequently in males) [66] such that it can be inferred that the suicide rate increase was related to charcoal burning suicide. 

In Singapore, before 1960–1964, hanging was the most commonly used suicide method. However, from 1980–1984, jumping had become the leading suicide method since then [67]. Due to the increasing availability of high-rise buildings during the period, the proportion of suicide by jumping increased from 18 % in 1960–1964 to 74% in 2000–2004 [55]. The suicide rate has been relatively stable (9.8–13.0/100,000) over the past five decades [68], although it fluctuated slightly upward in the 1980s and downward since 1995 [69]. The stability may be due to the collaborated efforts of Singapore government and non-government organizations to manage suicide risk factors such as individual social, economic and mental health problems [70], also to reduce access to jumping points by removal of long, common balconies and placement of higher railing, plastic barriers, encouragement of window security and monitoring of those with high suicide risk in families, and finally to break the falls by net-structures [71]. 

In Sri Lanka, the suicide rate by pesticide poisoning diminished after the ban of class I pesticides methamidophos and monocrotophos in 1995 and the further ban of class II pesticide endosulfan in 1998 [43]. Following the regulations, the rate of suicide by hanging gradually increased. The net effect is an overall decrease in suicide rate. Furthermore, advocacy has been underway for using specific lockable pesticide storage boxes to reduce pesticide accessibility in suicide attempts [3]. Further data collection is needed for assessing its effectiveness. 

In Thailand, since the late 1990s, the rate of female suicide by hanging became higher than by self-poisoning. In the period 2001–2005, ingestion of agricultural chemicals took the place of other substance intoxication and became the second leading suicide method [48,49]. Except for a mild upward fluctuation of suicide rate in 1998–2000 after the Asian financial crisis, suicide rate in Thailand has been relatively stable in 1977–2003 [48,72]. The introduction of a new method usually accompanied with an increase in suicide rate (such as the case in Hong Kong, Taiwan and Japan); the restriction of an existing method can potentially reduce suicide (such as Sri Lanka). In Singapore and Thailand, even though the composition of suicide methods changed over time, suicide rate remained stable, indicating that patterns of suicide in a country are shaped by multiple factors. 

### 3.3. Age and Gender Characteristics in Suicide Method Utilization

Information on age- and gender-specific methods of suicide was available in 14 countries/regions. 

#### 3.3.1. Age Characteristics

Suicide rates were, by and large, higher among older age groups in most parts of the World [23]. In India, elderly females preferred drowning and younger females preferred hanging [21]. However, hanging was still the most common method used by the elderly in India [16]. In China, regardless of age groups, pesticide poisoning was the most common method [73,74]. In Hong Kong, charcoal burning accounted for 18.3% of suicide; but 88.4% of the cases involved was in their middle age (25–59) [75]. The observation is very similar in Taiwan, where charcoal burning was most commonly observed in middle aged group [3]. In Iran, most of the people who self-immolated were young (mean age = 29) [26]. In South Korea and Taiwan, the youth were more likely to commit suicide by falling from a height [33,45]. In Saudi Arabia, people aged 20–49 occupied the majority of hanging cases [76]. 

#### 3.3.2. Gender Characteristics

There was widening of male-to-female suicide rate ratios in many Asian countries/regions in the past decade, including China, Taiwan, and Hong Kong [61,77]. In China, the excessive in female suicide rates is gradually disappearing after rapid urbanization and the concomitant decrease in pesticide self-poisoning among rural women [3]. The widened male-to-female suicide gender ratio in Taiwan and Hong Kong was due to the increased incidence of charcoal burning suicide among middle-aged men [61,77]. In South Korea, Taiwan, Japan and Singapore, a greater proportion of women committed suicide by falling from a height than that of men [28,42,45]. In Japan, a larger proportion of females preferred drowning than that of males [29,30]. In South Korea, men were more likely than women to commit suicide by hanging/suffocation [33]. In Singapore, those who jumped were more likely to be young females [42]. Self-immolation is a common method of suicide adopted by young females in India, Iran, Sri Lanka and Pakistan [21,26,78,79]. In India, a higher proportion of women chose hanging, self-immolation, or drowning compared with that of men [16,19,21]. Men preferred poisoning than women [18]. In Thailand, females used self-poisoning more frequently than males, who hanged themselves more frequently than females [49]. In Turkey, females preferred chemical ingestion to the use of firearms when committing suicide [50,52]. Elderly people preferred poisoning to firearms in suicide [53].

The narrower male-to-female suicide gender ratios found in many Asian countries/regions are generally due to higher suicide rates among women rather than lower rates in men. The higher rates among women are closely related to the adoption of highly lethal methods available in the household. For instance, pesticide poisoning in China, India, and Sri Lanka, building jumping in Hong Kong and Singapore, and ingestion of household chemicals in Turkey, Japan, South Korea, and Taiwan [80]. Kerosene, a flammable liquid used in self-immolation, is a common household substance for lighting and cooking in Iran, India, Iraq, and Sri Lanka [79,81,82,83]. Findings of the current study indicate that restricting access to these highly lethal suicide methods in the household can significantly reduce suicide rates in Asia, particularly among women.

Asian females tended to adopt lethal/ violent methods of suicide, such as jumping, drowning, hanging, and self-immolation. This is different from Western findings like that of Denning *et al.* (2000) [84], which females tended to use less lethal/violent suicide methods than men in the United States. The choice of suicide methods by Asian females may reflect their gender cultures and availability of suicide methods that were not compatible to their Western counterparts [29,56,85]. In addition, gender roles and individual preferences shaped by cultures may also contribute to gender differences in suicide across different cultures [86,87]. Suicidal females disliked corpse disfigurement and were concerned with how others might be shocked by it. Even though firearm suicide is common in many Western countries, the male to female suicide gender ratio for firearm suicide is very high (over 4 to 1), conversely, male to female suicide gender ratio for pesticide is much lower (usually less than 2 to 1) [80]. 

Overall, favored methods of suicide vary according to age and gender. For example, in China and Sir Lanka, self-poisoning by pesticide is commonly adopted by young women, while in many urbanized Asian cities/city states (e.g., Hong Kong, Singapore and Taipei City), young women prefer jumping from buildings [55,88]. In rapidly industrializing Asian countries like Taiwan and South Korea, pesticide self-poisoning is less common in young women. However, it is still prevalent in rural dwelling older women and men [80]. Like many parts of the World, hanging is still a common method of suicide in both genders in Asia [5,89]. Hence, restricting access to suicide methods may have different impacts on the suicide rate in different age and sex groups.

#### 3.3.3. Regional Findings

In China, the rural youth’s suicide method patterns were similar to that of the general population [90,91]. In Shanghai, one of the biggest cities in China, the leading suicide method in females was poisoning although in males, it was hanging [92]. In Penang Island, Malaysia, females preferred jumping to hanging; males did not differ between jumping and hanging [37]. In rural Sri Lanka, over half of the females committing suicide by self-poisoning were under 25 years old [93]. These local patterns were similar to the overall ones addressed above.

#### 3.3.4. Poisoning Trend

In China, rural young women’s relatively high suicide rate was especially related to pesticide use [37]. In both Taiwan and Hong Kong, charcoal burning was the dominant suicide method in middle-aged men in the past decade [61]. In Taiwan and Korea, the elderly were more likely to commit suicide by ingesting poisons/pesticides, whereas the youth were more likely to commit suicide by falling from a height [33,45]. In India, people younger than 44 tended to use poison [16]. In Japan, a greater proportion of males chose charcoal burning than that of females [30]. In Sri Lanka, over half of females who committed suicide by self-poisoning were younger than 25 years old and pesticide was the most common agent used in intentional self-poisoning [93]. However, for those who were younger than 20 years old, yellow oleander seeds were the most common agent used in intentional self-poisoning. For those above 20 with poisoning, pesticide was most often used [93]. Paraquat was used more frequently in suicides of the youth aged >20 years old [93]. In Thailand, females used self-poisoning more frequently than males [49]. 

## 4. Discussion

### 4.1. Dominant Methods of Suicide in Asia

The current review indicates that the dominant methods of suicide in Asia as a whole are hanging and solid/liquid poisoning (mainly with pesticides). There are unique patterns of suicide methods in Asian countries that markedly differ from those of the West. For example, building jumping is the most common method in Hong Kong and Singapore. This method accounts for a large portion of suicides in many populous cities in Asia, such as Taipei, Seoul, and Tokyo [55,88]. Charcoal burning suicide has become a popular method in Taiwan, Hong Kong, and Japan [3], while self-immolation, an uncommon suicide method in Western countries [79], is a common method in India, Iran, Pakistan, and Sri Lanka. Although not ranked high in the leading suicide methods, drowning and railway suicide were used in localities of India (9–16%) [18,21], Pakistan (11%) [39], Malaysia (10.9%) [37] and Japan (6–8%) [30]. Hanging is a common method in Asia, however, method restriction may not be plausible in preventing hanging, as ligature and ligation points are ubiquitous in our surroundings [89]. In addition to means restriction, suicide prevention may require social engineering to deal with life course stresses embedded in the current socio-cultural, economic, and religious contexts that trigger suicidal tendencies and facilitate the choices of different suicide methods in different stages of life. Any restriction of the method might also cause inconvenience to others. Raising awareness and removing the myths about suicide are very much needed in order to make the measures more receptive in the community.

The agents used in poisoning suicide in Asia differ prominently from those in Western countries. On top of pesticide poisoning, yellow oleander seeds used in Sri Lanka [93] and aluminium phosphide poisoning in some areas of India and Pakistan [6,22] are unheard of in Western countries. Variations of suicide methods within Asia are also prominent. For example, even though firearms are often regarded as a “Western method”, it is also a common method in Philippines, Saudi Arabia, and Turkey. The unique patterns reflect differences in *acceptability* and *availability *of the suicide method according to different socio-cultural, economic, and religious factors in these countries. A larger variety of common suicide methods warrants different approaches to suicide prevention, which should address these factors. 

### 4.2. Physical Availability and Socio-Cultural Acceptability of Suicide Means

Popular methods of suicide are not merely determined by easy availability of a specific method. Cultures and environments where suicidal persons live in contribute to the opportunities and limitations of what suicidal persons might use for committing suicide. For example, in places with high rise buildings, such as Hong Kong and Singapore, jumping suicide is the leading suicide method. In farming cultures such as rural China and Sri Lanka, pesticide suicide has been a dominant suicide method. 

Moreover, cultural factors imbue meanings to a given method, which account for its popularity. For example, suicide by self-immolation in many Asian countries reflects the cultural connotation of fire, and the use of fire symbolizes protest against injustices in life. Carrying the symbolic meaning of God and the purifier in Hinduism, fire is important in Hindu rituals and in prevalent ancient practices of sati (burning of the Hindu widow) and jauhar (women’s honorary self-immolation before their men go to battlefield) [23,79]. Concomitant with the easy access to kerosene, females in India tend to set themselves on fire during family disputes such as couple conflicts and dowry disputes. Aside from India, women in Muslim countries (such as Iran and Pakistan) also use domestic kerosene to burn themselves during heated family conflicts [78,81,94]. These findings suggest that for preventing self-immolation suicide, over and above restricting access to kerosene, socio-cultural interventions that empower women and engage religious leaders to be involved in prevention schemes are crucial. 

### 4.3. Popularization of New Methods and Cognitive Availability

In addition to traditional culture that influences method choice, the mass media as a powerful cultural channel has played a significant role in the dissemination of suicide and suicide methods [95]. In the case of charcoal burning suicide, there is a peculiar yet possibly hidden generalized pattern of transfer of knowledge and “technology” of suicide through mass media [96,97,98,99]. It is not that there is no case of suicide by gas poisoning before. The peculiar parts are how the gas (carbon monoxide) is produced and contained in the room (sealing of the windows and doors), how the images of suicide by charcoal burning (peaceful and intact bodies) have been depicted, and how the suicide method knowledge is being spread and received by people in these countries. Literature has shown how charcoal burning as a suicide method has spread in Taiwan, Hong Kong, and Japan through media and the internet [66,96,97,100,101]. With new social imaginations and meanings attached to it, this novel suicide method has attracted a group of people who did not have characteristics similar to those who used other suicide methods [96,98,102] To wit, it is likely that they have attracted people who might not otherwise have died by suicide and thus have increased suicide rates by novel cases of charcoal burning suicide.

A similar but less well known case is the spread of the knowledge of suicide by ingesting yellow oleander seeds through media reports in Sri Lanka [103]. This reveals that for suicide prevention targeting suicide methods, it is important to address both the restriction of access to the method and restriction of access to the knowledge of the method from the outset [8,9]. The extensive and sensational coverage of suicide death among the media in Asia is much more serious than that of western countries [104]. Some readers, especially the more vulnerable ones, may conceive that suicide by that specific method is a “normal” or “common” response to their personal problems. These people who have thought about suicide may intend to seek more information and understand the technical descriptions of some suicide methods. More than that, being exposed to web postings that encourage people to consider suicide may reinforce those vulnerable people to move from suicidal thought to action. So the Internet itself becomes a catalyst, and provides this group of people an interactive, privacy-protected and self-initiated medium to acquire the information they need and to reconfirm the “correctness” of their suicidal thought.

Self-regulation is suggested as an approach to reducing the potential harm of Internet suicide. But in the Web 2.0 era, the user-initiated media like applications of Wikipedia, blog or social networking have been growing rapidly every minute and these media are virtually impossible to regulate by the traditional modes, such as self-regulation by code of practice or licensing. However, if media and the internet do not abide by the suicide report guidelines, suicide prevention would be difficult unless the government is willing to enforce these guidelines. In view of the global nature of the Internet, it seems logical that a global effort rather than an Asian initiative is needed to contain the negative effect of Internet on suicides.

### 4.4. New Technologies and Suicide

From available data on trends in suicide methods, the introduction of new technologies (e.g., pesticides) and human constructions (e.g., high rise buildings) cause changes in the leading suicide methods in some Asian countries. In Sri Lanka and West Samoa, suicide by pesticide ingestion increased after the introduction of pesticides in agricultural practices [105]. In Singapore, the frequency of suicide by jumping increased parallel to the construction boom in high-rise buildings. There are other stories worthy of exploration as regards other technologies, such as kerosene, coal gas, highly lethal medication (e.g., tri-cyclic anti-depressants, barbiturates, and paracetamol), firearms, and others. Except for firearms that are intended for killing, harming or threatening others, these technologies are meant to improve people’s lives but unfortunately are used frequently in suicide. This phenomenon is a reminder of the importance of designing safety into new technologies with sufficient imagination on how people may use them for suicide. 

## 5. Conclusions

The epidemiologic characteristics and recent trends of common suicide methods in the 17 Asian countries/regions reflect specific socio-cultural, environmental, economic, and religious situations in the region. Common suicide methods shift with the introduction of technologies and constructions, and will affect age and sex subgroups differently. As the data reviewed repeatedly indicate that method availability as a major factor in method choice and likely as a factor that increases overall suicides, measures should be taken where possible to limit access to means of popular methods of suicide. Such measures should involve legal restrictions, regulation and/or safe storage of dangerous substances commonly used for suicide in the region in question, such as charcoal in Hong Kong and Taiwan and lethal pesticides in Sri Lanka and rural China. In the information society, suicide prevention that focuses on suicide methods must monitor and control the innovation and spread of knowledge and practices of suicide “technologies”. It may be more cost-effective to design safety into technologies as a way of suicide prevention while there is no rash of suicides yet by the new technologies. 

Research on suicide methods in Asia or even in western countries is still limited and sporadic. Without sound research evidence, unavailable will be a comprehensive and updated list of risk and protective suicide factors for designing suicide prevention programs. Considering the limitation of resources and large population in many Asian countries, it is more advisable to adopt public health approaches to suicide prevention with sensitivity to socio-cultural, economic, and religious factors in different countries. In some countries, socio-cultural interventions that empower women and engage religious leaders to be involved in prevention schemes are crucial. 

Indeed, policy-making as regards suicide prevention may be an important step in advancing safer and more cost-effective technologies. It is necessary to think about how much loss of life can be justified by the benefits brought about by these technologies and when not dispensable, how to offer timely and effective treatment and even compensation for suicides that used these technologies. There are many challenges ahead and ample opportunities to make suicide prevention work and work well in Asia. 
